# Spectrophotometric Determination of Pipazethate HCl and Dextromethorphan HBr using Potassium Permanganate

**Published:** 2008-12

**Authors:** Ayman Abou El-Fetouh Gouda, Ragaa El-Sheikh, Zeineb. El Shafey, Nagda. Hossny, Rham El-Azzazy

**Affiliations:** 1 *Chemistry Department, Faculty of Science, Zagazig University, Zagazig, Egypt;*; 2 *Chemistry Department, Faculty of Science (Girl’s), Al-Azhar University, Cairo, Egypt*

**Keywords:** spectrophotometry, pipazethate HCl, dextromethorphan HBr, potassium permanganate, oxidation reactions, pharmaceutical formulations

## Abstract

Rapid, simple and sensitive validated spectrophotometric methods have been described for the assay of pipazethate HCl (PiCl) and dextromethorphan HBr (DEX) either in pure form or in pharmaceutical formulations. The proposed methods were based on the oxidation of the studied drugs by a known excess of potassium permanganate in acidic medium and estimating the unreacted permanganate with amaranth dye (method A), acid orange II (method B), indigocarmine (method C) and methylene blue (method D), in the same acid medium at a suitable λ_max_=521, 485, 610 and 664 nm, respectively. Beer’s law is obeyed in the concentration range of 2.0-16 and 2.0-15 μg mL^-1^ for PiCl and DEX, respectively with correlation coefficient (*n*=6) ≥ 0.9993. The apparent molar absorptivity and sandell sensitivity values are in the range 1.062-1.484 × 10^4^, 3.35-4.51 × 10^4^ L mol^-1^ cm^-1^ and 29.36-41.03, 8.21-11.06 ng cm^-2^ for PiCl and DEX, respectively. Different variables affecting the reaction were studied and optimized. The proposed methods were applied successfully to the determination of the examined drugs either in a pure or pharmaceutical dosage forms with good accuracy and precision. No interferences were observed from excipients and the results obtained were in good agreement with those obtained using the official methods.

## INTRODUCTION

Pipazethate hydrochloride (PiCl), 10*H*-pyrido[3,2-*b*][1,4]benzothiadiazine-10-carboxylic acid 2-(2-piperidinoethoxy)ethyl ester (1) is a bronchodilator that suppresses irritative and spasmodic cough by inhibiting the excitability of the cough center and the peripheral neural receptors in the respiratory passage. The response to the drug takes about 10–20 min and lasts for 4–6 h (Scheme I). Pipazethate has been determined using a limited number of techniques including; spectrophotometry (2-6), TLC (7), HPLC (8), Conductimetry (9) and ISE (10). PiCl was used in determination of Mo (VI) in alloy steels and soil samples (11).

**Figure 1:**
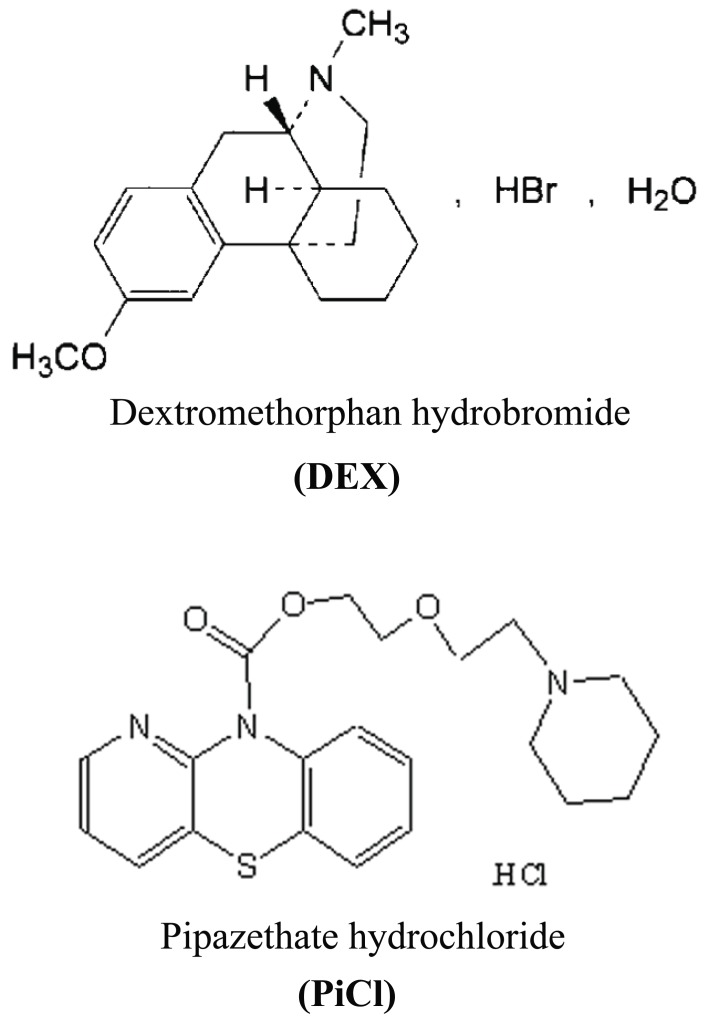
The chemical structure of the studied drugs.

Dextromethorphan hydrobromide (DEX), [(+)-3-Methoxy-17-methyl-9α, 13α, 14α-morphinan hydrobromide monohydrate] is a cough suppressant, used for the relief of non-productive cough; it has a central action on the cough centre in the medulla (12) (Fig. 1). Different methods reported for the determination of DEX in bulk drug, in dosage forms with other drugs in cough-cold products and in biological samples. HPLC have been reported (13, 14), spectrophotometry (4, 5), first and second-derivative technique UV-spectrophotometry (15-18), capillary electrophoresis (19, 20), GC (21) and LC (22, 23).

The purpose of the present work is the development of a simple spectrophotometric method for the determination of anti-tussive drugs; pipazethate hydrochloride (PiCl) and dextromethorphan hydrobromide (DEX) in bulk and in their pharmaceutical formulations, based on the discoloring redox reaction with an excess of KMnO_4_, used as self indicator and the determination of unreacted oxidant by the decrease in absorbance of the dyes; amaranth dye (AM) (method A), acid orange II (AO) (method B), indigocarmine (indigo) (method C) and methylene blue (MB) (method D).

## EXPERIMENTAL

### Materials

Pure pipazethate HCl (PiCl) and dextromethorphan HBr (DEX) were obtained from the Egyptian International Pharmaceutical Industries Company (EIPICO). All pharmaceutical preparations were obtained from commercial sources in the local market.

### Reagent

All chemicals used were of analytical grade and all solvents were for spectroscopic grade.

An a stock aqueous solution of 1.0 × 10^-3^ M of amaranth (AM) (E-Merck, Germany), acid orange II (AO) (E-Merck, Germany), indigocarmine (Aldrich), and methylene blue (MB) (E-Merck, Germany) were prepared by dissolving an accurate weight of dye in least amount of water and completed to the mark in a 100 mL calibrated flask with water. A 5.0 × 10^-4^ M is prepares by diluting appropriate volume of the previously prepared solution. The stock solutions of dyes were allowed to stand at room temperature for a few weeks without any significant decay.

A stock solution of 5.0 × 10^-3^ M KMnO_4_ (Aldrich) was prepared by dissolving 0.079 g of KMnO_4_ (Aldrich) in 10 mL of warm bidistilled water then completed to the mark in a 100 mL calibrated flask. Standardized using sodium oxalate (24) and kept in a dark bottle. A 5.0 × 10^-4^ M solution of KMnO_4_ was prepared by diluting the previously stock solution with water and 2.0 M H_2_SO_4_ was prepared.

### Apparatus

All the absorption spectral measurements were made using Kontron 930 (UV-Visible) spectrophotometer (German) with scanning speed 200 nm/min, and band width 1.0 nm equipped with 10 mm matched quartz cells.

### Standard Solutions

Stock solutions (100 μg mL^-1^) of the studied drugs were freshly prepared daily by dissolving 10 mg of the drug in distilled water and then, completed to the mark in a 100 mL calibrated flask with distilled water. Working standard solutions were prepared by suitable dilution of the stock.

## METHOD

### Recommended procedures

Pipette a 1.0 mL aliquot of the examined drugs solution (100 μg mL^-1^) into a series of 10 mL calibrated flasks, followed by acidification by adding 0.5 mL of 2.0 M H_2_SO_4_. Two mL and 1.5 mL of 5.0 × 10^-4^ M KMnO_4_ were added and heated in a water bath at 60 ± 2°C for 5.0 min and 10 min with PiCl and DEX, respectively. The mixture was cooled to laboratory temperature, then 1.0, 1.5, 1.2 and 2.0 mL of (5.0 × 10^-4^ M) of AM, AO Indigo and MB, respectively with PiCl and DEX. The volume was completed to 10 mL with water. The decrease in color intensities were measured spectrophotometrically at their corresponding maximum wavelengths λ_max_ values, 521, 485, 610 and 664 nm for AM, AO, Indigo and MB, respectively. The concentration of each drug was determined from a calibration graph constructed under the same conditions.

### Applications for pharmaceutical formulations

**Procedure for tablets.** The contents of twenty tablets of (Selgon tablets, 20 mg PiCl per tab. or Tussilar tablets, 10 mg DEX per tab.), were crushed powdered, weighed out and the average weight of one tablet was determined. An accurate weight equivalent to 10 mg of pure drug was dissolved in 20 mL distilled water and then filtered. The filtrate was diluted to 100 mL with distilled water in a 100 mL calibrated flask. This solution was further diluted stepwise to the request concentration with water and then analyzed by the recommended procedure.

**Procedure for drops.** The contents of five bottles (Selgon drops, 40 mg PiCl per mL or Tussilar drops, 1.0 g DEX per 15 mL) were mixed and the average volume for one bottle was determined. An aliquot of the solution equivalent to 10 mg PiCl and DEX was quantitatively transferred to 100 mL calibrated flask and made up to the mark with bidistilled water. The above stated procedures described were applied to determine drug concentrations.

## RESULTS AND DISCUSSION

The optimum conditions for color development for each method were established by varying the parameters one at a time, keeping the others fixed and observing the effect produced on the absorbance of the colored species.

### Absorption spectra

**Figure 2:**
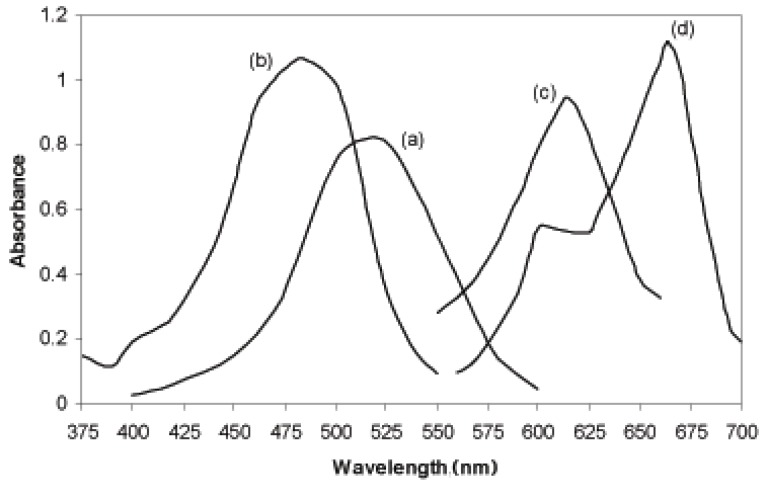
Absorption spectra of the oxidation product between 8.0 μg mL^-1^ PiCl, KMnO_4_ and, (a) AM, (b) AO, (c) Indigo and (d) MB by heating at 60 ± 2°C for 5.0 min.

The spectrophotometric method for the determination of PiCl and DEX is based on their oxidation with a known excess of KMnO_4_ in acidic medium and subsequent determination of residual oxidant by reacting it with fixed amount of amaranth dye (AM), acid orange II (AO), indigocarmine (indigo) and methylene blue (MB) show characteristics λ_max_ values at 521, 485, 610 and 664 nm for AM, AO, indigo and MB methods, respectively (Fig. 2).

### Effect of heating time

**Figure 3:**
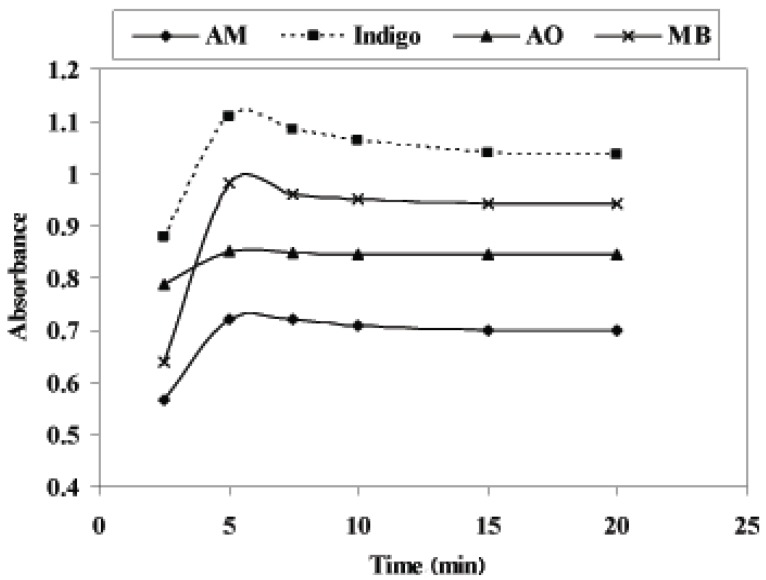
Effect of heating time on the oxidation of 8.0 μg mL^-1^ PiCl-dye at the optimum wavelengths λ_max_ of each dye.

In order to obtain the highest and most stable absorbance, the effect of heating time on the oxidation reaction of PiCl and DEX was catalyzed by heating in a water bath at 60 ± 2°C for the periods ranging for 2.5-20 min. the time required to complete the reaction and maximum absorbance was obtained after 5.0 min for PiCl and 10 min for DEX. After oxidation process, the solution must be cooled at least for 3.0 min before addition of dye (Fig. 3).

### Effect of oxidant concentration

**Figure 4:**
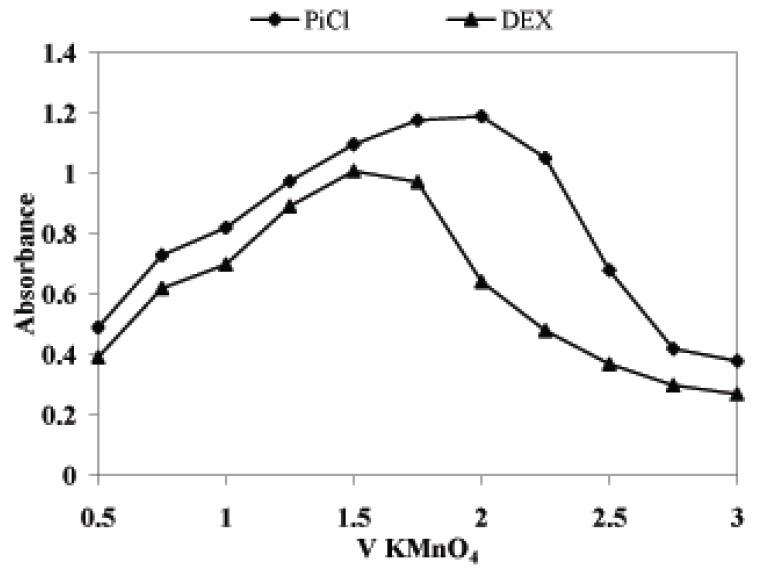
Effect of volume of 5.0 × 10^-4^ M KMnO_4_ on the development of the reaction product: 8.0 μg mL^-1^ DEX with MB and 10 μg mL^-1^ PiCl with AO.

When a study on the effect of KMnO_4_ on color development was performed, it was observed that in both cases the absorbance increased with increase in the volume of KMnO_4_ (5.0 × 10^-4^ M). It reached maximum when 2.0 ml and 1.5 ml of KMnO_4_ solution was added to a total volume of 10 ml for PiCl and DEX, respectively. The color intensity decreased above the upper limits. Therefore, 2.0 ml and 1.5 ml of KMnO_4_ were used for all measurements (Fig. 4).

### Effect of acid concentration

**Figure 5:**
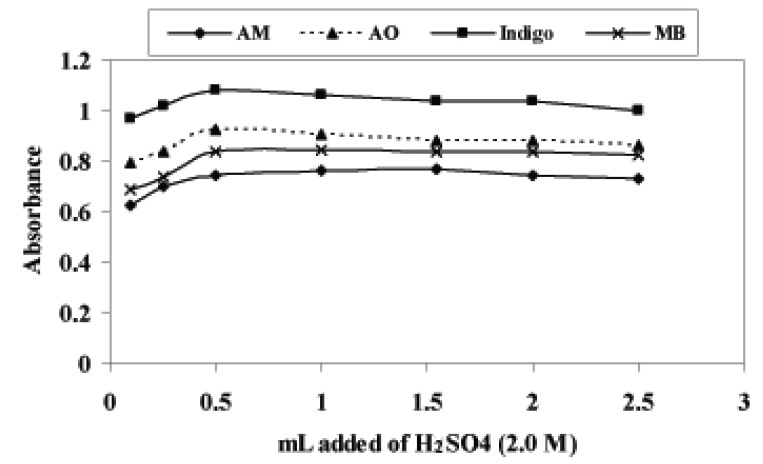
Effect of mL added of Sulfuric acid (2.0 M) on absorbance of PiCl with (5.0 × 10^-4^ M) KMnO_4_ and dyes (5.0 × 10^-4^ M).

To study the effect of acid concentration, different types of acids were examined (H_2_SO_4_, H_3_PO_4_ and CH_3_COOH) to achieve maximum yield of redox reaction. The results indicated that the sulphuric acid was the preferable acid with KMnO_4_ as oxidant. The reaction was performed in a series of 10 mL volumetric flask containing 8.0 μg mL^-1^ of the cited drugs, different volumes (0.1–2.5 mL) of 2.0 M H_2_SO_4_ and 2.0 and 1.5 mL of KMnO_4_ (5.0 × 10^-4^ M) with PiCl and DEX, respectively were added. After 5.0 min for PiCl and 10 min for DEX heating time at 60 ± 2°C in a water bath, the solution was cooled for about 3.0 min; the dyes (1.0, 1.5, 1.2 and 2.0 mL of AM, AO, indigo and MB, respectively) were added, then complete to 10 mL total volume with water. It was found that the maximum absorbance was obtained at 0.5 mL of 2.0 M H_2_SO_4_. Above this volume, the absorbance decreased for PiCl, where as for DEX the absorbance remained constant. Therefore, a volume of 0.5 ml of 2.0 M H_2_SO_4_, was used for all measurements (Fig. 5).

### Effect of dye concentration

**Figure 6:**
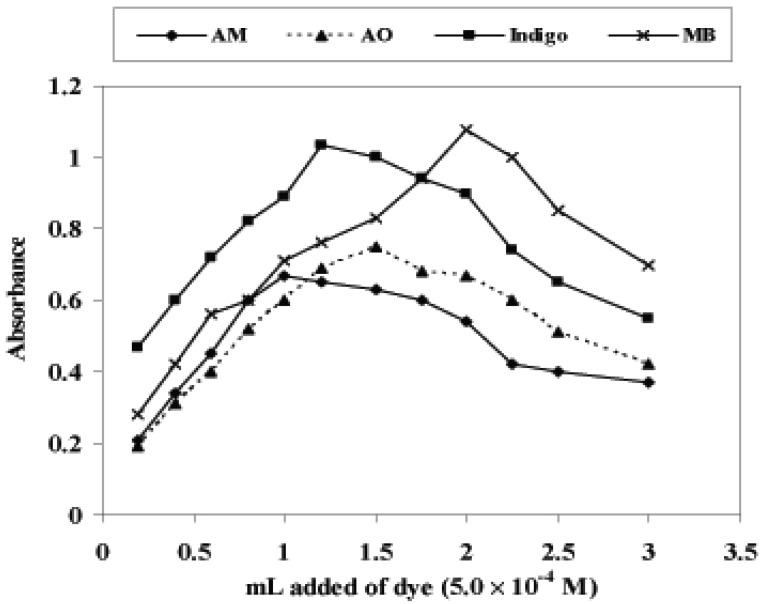
Effect of added dyes (5.0 × 10^-4^ M) on absorbance of 10 μg mL^-1^ of PiCl with KMnO_4_ (5.0 × 10^-4^ M).

In order to ascertain the linear relationship between the volume of added KMnO_4_ and the decrease in absorbance of AM, AO, Indigo and MB, experiments were performed using 0.5 mL of 2.0 M H_2_SO_4_ with varying volumes of KMnO_4_. The decrease in absorbance was found to be linear up to 2.0 and 1.5 mL of 5.0 × 10^-4^ M KMnO_4_ with optimum volumes (1.0, 1.5, 1.2 and 2.0 mL of AM, AO, indigo and MB, respectively) for 8.0 μg mL^-1^ of PiCl and DEX (Fig. 6). The color was found to be stable up to 24 h.

### Stoichiometric ratio

**Figure 7:**
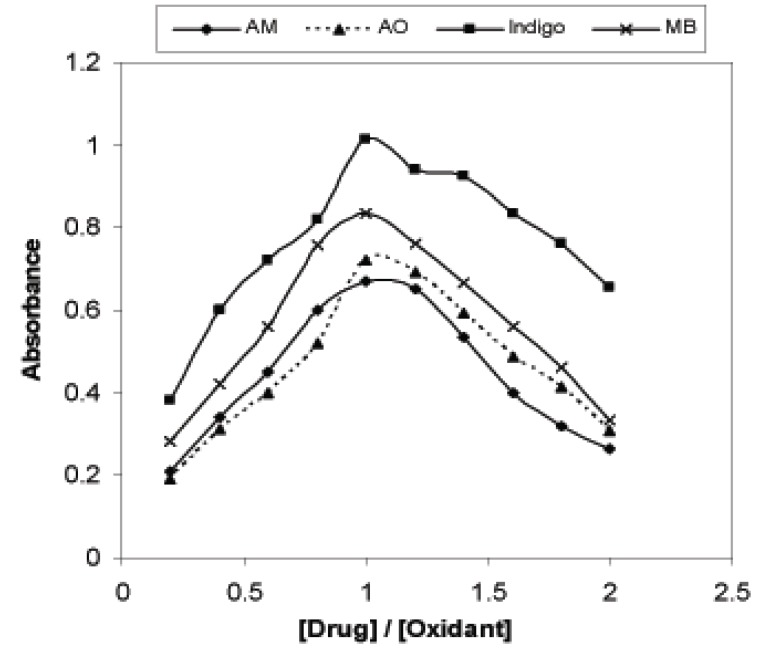
Continuous variations graph for the reaction between 5.0 × 10^-4^ M PiCl and 5.0 × 10^-4^ M KMnO_4_ with dyes (5.0 × 10^-4^ M).

Job’s method of continuous variation and the molar ratio method described by Yoe and Jones (25, 26), was employed to determined the stoichiometry of drug, oxidant and dyes. Keeping the sum of the molar concentration of both fixed, the ratio of the concentrations of each two in the mixture was varied and the absorbances of the mixture were recorded at the suitable wavelength against reagent blank. The maximum absorbance corresponds to the stoichiometric ratio. Stoichiometric ratio was found to be 1:1 for drug to oxidant; drug to dyes and oxidant to dyes as shown in (Fig. 7) (Table 1).

**Table 1:** Analytical parameters and optical characteristics of the proposed methods with PiCl and DEX

Parameters	PiCl				DEX			
	AM	AO	Indigo	MB	AM	AO	Indigo	MB

λ_max’_ (nm)	522	610	664	485	520	610	664	485
Beer’s law linits (μg mL^-1^)	2.0-16	2.0-14	2.0-14	2.0-10	2.0-10	4.0-12	4.0-15	4.0-10
Ringbom Limits (μg mL^-1^)	2.5-15	3.0-13	3.0-12.5	2.5-9.5	3.0-9.0	5.0-10.5	5.0-14	5.5-9.5
Molar absorpitivity × 10^4^ (L mol^-1^ cm^-1^)	1.062	1.193	1.37	1.484	4.2	3.35	3.72	4.51
Sandell sensitivity (ng cm^-2^)	41.03	36.52	31.80	29.36	8.82	11.06	9.96	8.21
Regression equation^a^								
Slope (b)	0.0497	0.0571	0.0678	0.0661	0.1812	0.21	0.168	0.118
Intercept (a)	0.0051	0.0003	-0.0137	0.014	-0.446	-0.742	-0.521	-0.736
Correlation coefficient (*r*)	0.9999	0.9999	0.9997	0.9998	0.9990	0.9991	0.9994	0.9995
S_y/x_	0.3118	0.2737	0.3384	0.2687	0.331	0.380	0.308	0.361
SD of slope (*S_b_*)	0.2647	0.234	0.32	0.2317	0.105	0.120	0.097	0.114
SD of intercept (*S_a_*)	0.0249	0.0261	0.0339	0.0469	1.64	2.15	1.96	2.05
Detection limits (μg mL^-1^)	0.30	0.16	0.21	0.18	0.09	0.26	0.37	0.21
Quantification limit (μg mL^-1^)	0.99	0.53	0.69	0.60	0.30	0.87	1.23	0.70
RSD^b^ %	0.79	0.83	0.68	0.54	0.92	1.04	0.58	0.79
*t*^c^	0.122	0.18	0.118	0.206	0.091	0.791	0.618	0.301
*F*^c^	1.895	1.756	2.394	1.28	1.058	2.40	1.435	1.03

a A = a + b C, where C is the concentration in μg mL^-1^;

b Average of six determinations;

c Calculated *t*- and *F*-value; tabulated *t* and *F*-value for five degrees of freedom; p=0.05 are 2.57 and 5.05.

### Validation of the proposed methods

**Linearity.** At described experimental conditions for PiCl and DEX determination, standard calibration curves for PiCl and DEX with KMnO_4_ and dyes, were constructed by plotting absorbance’s vs. concentrations. The statistical parameters were given in the regression equation calculated from the calibration graphs, along with the standard deviations of the slope (*S_b_*) and the intercept (*S_a_*) on the ordinate and the standard deviation residuals (*S_y/x_*).

The linearity of calibration graphs was proved by the high values of the correlation coefficient (*r*) and the small values of the y-intercepts of the regression equations. The apparent molar absorptivities of the resulting colored ion-pair complexes and relative standard deviation of response factors for each proposed spectrophotometric method were also calculated and recorded in Table 1. The molar absorptivity of D>C>B>A method for PiCl, while for DEX the molar absorptivity of D>A>C>B method.

**Sensitivity.** The detection limits (LOD) for the proposed methods were calculated using the following equation (27):

LOD = *3s / k*

where *s* is the standard deviation of replicate determination values under the same conditions as for the sample analysis in the absence of the analyte and *k* is the sensitivity, namely the slope of the calibration graph. In accordance with the formula, the detection limits were found to be 0.30, 0.16, 0.21 and 0.18 μg mL^-1^ for A, B, C and D methods, respectively. Whereas; for DEX the detection limits were found to be 0.09, 0.26, 0.37 and 0.21 μg mL^-1^ for A, B, C and D methods, respectively.

The limits of quantitation, LOQ, defined as (27);

LOQ = *10 s / k*

According to this equation, the limit of quantitation were found to be 0.99, 0.53, 0.69 and 0.60 μg mL^-1^ for A, B, C and D methods, respectively. Whereas; for DEX the detection limits were found to be 0.30, 0.87, 1.23 and 0.70 μg mL^-1^ for A, B, C and D methods, respectively.

**Specificity, Precision, and Accuracy.** Specificity of Oxidation-reduction reaction and selective determination of PiCl and DEX with KMnO_4_ and dyes could be possible. Percentage relative standard deviation (RSD %) as precision and percentage relative error (Er %) as accuracy of the suggested method were calculated. Precision was carried out by six determinations at four different concentrations in these spectrophotometric methods. The percentage relative error calculated using the following equation:

Er % = [(founded – added) / added] × 100

The inter-day precision and accuracy results are shown in (Table 2). These results of accuracy and precision show that the proposed methods have good repeatability and reproducibility.

**Table 2:** Evaluation of accuracy and precision data for PiCl and DEX obtained by the proposed methods

Method	PiCl				DEX			
	Added (μg mL^-1^)	Recovery %	Precision RSD%^a^	Accuracy Er %	Taken (μg mL^-1^)	Recovery %	Precision RSD%^a^	Accuracy Er %

A	4.0	100.1	0.84	0.10	2.0	100.09	0.68	0.09
	8.0	99.57	0.92	-0.425	4.0	99.92	0.90	-0.08
	12	99.95	0.77	-0.05	6.0	99.70	0.88	-0.30
	16	100.2	1.08	0.20	8.0	100.15	1.17	0.15
B	4.0	99.72	0.99	-0.275	4.0	99.20	0.76	-0.80
	6.0	99.15	0.85	-0.85	6.0	100.20	0.57	0.20
	8.0	98.94	1.12	-1.06	8.0	99.90	0.66	-0.10
	10	99.45	0.88	-0.55	10	100.31	0.39	0.31
C	3.0	100.12	0.76	0.133	4.0	99.70	1.08	-0.30
	6.0	99.85	0.79	-0.15	6.0	98.92	0.54	-1.08
	9.0	99.92	0.81	-0.078	9.0	99.30	0.45	-0.70
	12	100.15	1.23	0.15	12	100.10	0.61	0.10
D	2.0	100.07	1.06	0.07	4.0	100.67	0.57	0.67
	4.0	99.25	1.42	-0.75	5.0	100.50	0.72	0.50
	6.0	101.17	0.96	1.17	6.0	99.78	0.69	-0.22
	8.0	99.83	0.88	-0.17	8.0	100.08	0.94	0.083

a Mean of six determination. RSD%, percentage relative standard deviation; Er%, percentage relative error.

**Robustness and Ruggedness.** For the evaluation of method robustness, some parameters were interchanged; KMnO_4_ concentration, dye concentration, wavelength range, and heating time. The capacity remained unaffected by small deliberate variations. Method ruggedness was expressed as RSD % of the same procedure applied by two analysts and in two different instruments on different days. The results showed no statistical differences between different analysts and instruments suggesting that the developed methods were robust and rugged.

### Interferences

In pharmaceutical analysis, it is important to test the selectivity towards excipients and additives added to the pharmaceutical preparations of PiCl and DEX. It is clear from the results obtained for the pharmaceutical preparations that the commonly encountered excipients such as starch, talc, glucose, alginate and stearate did not interfere indicating a high selectivity for determining the studied PiCl and DEX in its dosage forms.

### Pharmaceutical applications

The proposed methods were successfully applied to determine the drugs studied PiCl and DEX in tablets and drops. Six replicate determinations were made. Moreover, to check the validity of the proposed methods, dosage forms were tested for possible interference with standard addition method. There was no significant difference between slopes of calibration curves and standard addition methods at four methods. Therefore it is concluded that the excipients in pharmaceutical dosage forms of PiCl and DEX such as starch, lactose, glucose, sugar, talc, sodium chloride, titanium dioxide, and magnesium stearate were not found any interference in the analysis of PiCl and DEX. The results were compared statistically by student’s t- test (for accuracy) and variance ratio F- test (for precision) with official methods at 95% confidence level with five degrees of freedom (Table 3, 4). The results showed that the *t* and *F*- values were less than the critical value (27) indicating that there were no significant differences between the proposed and official methods. Because the proposed methods were more reproducible with high recoveries they can be recommended for routine analysis in majority of drug quality control laboratories.

**Table 3:** Application of the standard addition technique for the determination of PiCl in dosage forms using the proposed methods

Method	Taken (μg mL^-1^)	Selgon tablets (20 mg/tab.)			Selgon drops (40 mg/mL)		
		Added (μg mL^-1^)	Recovery^a^ %	Reference method	Added (μg mL^-1^)	Recovery^a^ %	Reference method

A	4	--	99.80		--	97.85	
		2.0	100.50		2.0	99.50	
		4.0	100.35		4.0	101.00	
		6.0	100.20		6.0	100.25	
		8.0	99.65		8.0	100.60	
		10	100.80		10	99.25	
	Mean^a^ ± SD (p=0.05)		100.22 ± 0.832	99.70 ± 1.16		100.08 ± 0.67	100.50 ± 0.63
	*t*^b^		0.364			0.457	
	*F*^b^		1.944			1.13	
B	2	--	99.51		--	98.95	
		2.0	99.96		2.0	99.50	
		4.0	100.01		4.0	100.40	
		6.0	99.10		6.0	100.55	
		8.0	99.85		8.0	99.50	
		10	100.30		10	99.90	
	Mean^a^ ± SD (p=0.05)		99.97 ± 0.94	99.70 ± 1.16		99.80 ± 0.61	100.50 ± 0.63
	*t*^b^		0.181			0.798	
	*F*^b^		1.52			1.07	
C	2	--	99.60		--	99.35	
		2.0	100.45		2.0	99.70	
		4.0	100.15		4.0	100.40	
		6.0	100.30		6.0	99.20	
		8.0	99.85		8.0	100.50	
		10	99.60		10	99.75	
	Mean^a^ ± SD (p=0.05)		99.99 ± 0.86	99.70 ± 1.16		99.82 ± 0.53	100.50 ± 0.63
	*t*^b^		1.444			0.826	
	*F*^b^		1.82			1.413	
D	1.0	--	99.89		--	99.25	
		1.0	99.95		1.0	100.40	
		2.0	100.40		2.0	99.50	
		4.0	100.05		4.0	100.70	
		6.0	100.20		6.0	100.25	
		8.0	99.75		8.0	100.60	
	Mean^a^ ± SD (p=0.05)		100.04 ± 1.232	99.70 ± 1.16		100.12 ± 0.601	100.50 ± 0.63
	*t*^b^		0.201			0.436	
	*F*^b^		1.128			1.10	

a Average of six determinations;

b Calculated *t*- and *F*-value; tabulated *t* and *F*-value for five degrees of freedom; *p*=0.05 are 2.57 and 5.05.

**Table 4:** Application of the standard addition technique for the determination of DEX in dosage forms using the proposed methods

Method	Taken (μg mL^-1^)	Tussilar tablets (10 mg/tab.)			Tussilar drops (1.0 g/15 mL)		
		Added (μg mL^-1^)	Recovery^a^ %	Reference method	Added (μg mL^-1^)	Recovery^a^ %	Reference method

A	4	--	99.80		--	97.85	
		2.0	100.50		2.0	99.50	
		4.0	100.35		4.0	101.00	
		6.0	100.20		6.0	100.25	
		8.0	99.65		8.0	100.60	
		10	100.80		10	99.25	
	Mean^a^ ± SD (p=0.05)		100.22 ± 0.432	99.92 ± 0.85		100.08 ± 0.67	100.18 ± 0.81
	*t*^b^		0.026				
	*F*^b^		1.106				
B	2	--	99.51		--	98.95	
		2.0	99.96		2.0	99.50	
		4.0	100.01		4.0	100.40	
		6.0	99.10		6.0	100.55	
		8.0	99.85		8.0	99.50	
		10	100.30		10	99.90	
	Mean^a^ ± SD (p=0.05)		99.97 ± 0.423	99.92 ± 0.85		99.80 ± 0.61	100.18 ± 0.81
	*t*^b^		0.296			0.798	
	*F*^b^		1.27			1.07	
C	2	--	99.60		--	99.35	
		2.0	100.45		2.0	99.70	
		4.0	100.15		4.0	100.40	
		6.0	100.30		6.0	99.20	
		8.0	99.85		8.0	100.50	
		10	99.60		10	99.75	
	Mean^a^ ± SD (p=0.05)		99.99 ± 0.36	99.92 ± 0.85		99.82 ± 0.53	100.18 ± 0.81
	*t*^b^		0.09			0.826	
	*F*^b^		1.43			1.413	
D	1.0	--	99.63		--	100.04	
		1.0	99.91		1.0	99.50	
		2.0	100.16		2.0	100.40	
		4.0	99.10		4.0	100.55	
		6.0	99.85		6.0	99.95	
		8.0	100.20		8.0	99.90	
	Mean^a^ ± SD (p=0.05)		99.81 ± 0.41	99.92 ± 0.85		100.06 ± 0.38	100.18 ± 0.81
	*t*^b^		0.302			0.436	
	*F*^b^		1.067			1.10	

a Average of six determinations;

b Calculated *t*- and *F*-value; tabulated *t* and *F*-value for five degrees of freedom; *p*=0.05 are 2.57 and 5.05.

### Chemistry of colored species

**Figure 8:**
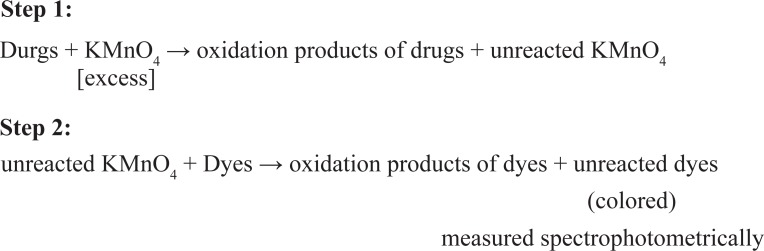
The possible sequences of Oxidation-reduction reaction.

The proposed methods are based on the oxidation of the cited drugs by excess of KMnO_4_ to form oxidation products besides unreacted KMnO_4_ (step 1), and followed by the determination of unreacted KMnO_4_ by measuring the decrease in the absorbance of AM, AO, Indigo and MB dyes at their λ_max_ (step 2). The possible sequences of reactions are presented in Fig. 8.

## CONCLUSION

The order of λ_max_ values among the proposed methods for the determination of the cited drugs is D>C>A>B. The higher λ_max_ of the visible spectrophotometric methods over reported UV and visible spectrophotometric methods is decisive and advantageous since interference from the excipients should be far less at higher wavelengths. The proposed methods are accurate and precise as indicated by good recoveries of the drugs and low RSD values. The proposed methods can be applied for routine analysis and in quality control laboratories for quantitative determination of the cited drugs both in the pure and dosage forms.
